# Association and implications in triple negative and triple positive breast cancer: Relationship with sociodemographic and reproductive factors in Pakistan

**DOI:** 10.12669/pjms.346.15763

**Published:** 2018

**Authors:** Faria Fatima, Abdul Hameed, Ghulam Haider, Sitwat Zehra, Abid Azhar, Saima Saleem

**Affiliations:** 1 *Faria Fatima, Research Fellow, The Karachi Institute of Biotechnology and Genetic Engineering (KIBGE), University of Karachi, Karachi, Pakistan*; 2 *Abdul Hameed, Ph.D. Institute of Biomedical and Genetic Engineering (IB&GE), Islamabad, Pakistan*; 3 *Ghulam Haider, MBBS, FCPS (Medicine), FCPS (Oncology) Jinnah Postgraduate Medical Centre (JPMC), Karachi, Pakistan*; 4 *Sitwat Zehra, Ph.D. The Karachi Institute of Biotechnology and Genetic Engineering (KIBGE), University of Karachi, Karachi, Pakistan*; 5 *Abid Azhar, Ph.D., D.Sc. The Karachi Institute of Biotechnology and Genetic Engineering (KIBGE), University of Karachi, Karachi, Pakistan*; 6 *Saima Saleem, Ph.D. The Karachi Institute of Biotechnology and Genetic Engineering (KIBGE), University of Karachi, Karachi, Pakistan*

**Keywords:** Triple Negative, Triple Positive, Parity, Child bearing, Breast Cancer

## Abstract

**Background & Objectives:**

Triple negative and triple positive breast cancer have adverse effects than other types of breast cancer. However, triple negative has poor prognosis with short survival as compared with triple positive breast cancer. Good prognosis is one of the key factors for successful treatment trial. This study aimed to find out the association of sociodemographic and reproductive features like parity, menopause, number of child bearing as risk factors in the development and prognosis of triple negative and triple positive breast cancer.

**Methods:**

This study is a part of an ongoing project which is being conducted in Karachi from 2013 to 2020. Informed consent from triple negative breast cancer (n=134) and triple positive breast cancer (n=87) patients were taken prior to their recruitment into the study. Demographic, anthropometric, reproductive and disease history of patients were recorded. Means, frequency distribution, data classification and association analyses were done by SPSS version 17.0.

**Results:**

Statistical analyses revealed that delayed first child bearing age and lower number of children are associated with the development of triple negative breast cancer. However, no significant effect of these parameters has been observed on the outcomes of triple positive breast cancer.

**Conclusions:**

Reproductive factors have more pathological implications than sociodemographic factors in both triple positive and triple negative breast cancer development. These findings might prove to be beneficial for effective and better breast cancer management.

## INTRODUCTION

As stated by World Health Organization (WHO), worldwide breast cancer incidence accounts for 11.9%. Every year about 1.67 million women are diagnosed with breast cancer around the world.^1^ Out of every nine women one suffers from breast cancer in Pakistan. It has the incidence of 23%.^2^,^3^

Evidence from epidemiological data have shown that demographic and social factors are related to the late diagnosis of breast cancer. Those sociodemographic factors include age, ethnicity, marital status, consanguinity and family history. Cultural shyness, inadequate health awareness and health care access have been proposed to be one of the major reasons for the association of these factors with breast cancer.^4^-^6^ Clinical, anthropometric, reproductive features and other related characteristics have a huge impact on long term prognosis of breast cancer development and treatment.^7^

Higher body mass index (BMI) increase the risk of breast cancer development. Some of the reproductive features reduce the risk of breast cancer development. Parity, multiparous reproductive lifestyle, high number of children/full term pregnancies are some of the reproductive features which shield and reduce the susceptibility towards breast cancer.^4^,^7^,^8^

Development of hormonal therapies improve the management of triple positive breast cancer over triple negative breast cancer. Treatment and prognosis of triple negative is poor and difficult with low survival rate.^9^

This study aimed to find out the impact of sociodemographic, anthropometric and reproductive factors on the pathogenesis of triple positive and triple negative breast cancer.

## METHODS

It is a cross sectional study for the demographic risk factors of breast cancer and it is a part on of ongoing project which is being conducted in Karachi from 2013 to 2020. All the procedures followed, were approved by institutional review board of The Karachi Institute of Biotechnology and Genetic Engineering (KIBGE) and Jinnah Postgraduate Medical Center (JPMC). Prior to the recruitment, informed consent was taken from all the subjects.

### Subject Selection

Total 134 triple negative breast cancer (Estrogen, Progesterone and Her2neu negative) and 87 triple positive breast cancer (estrogen, progesterone and Her2neu positive) cases were included in this study. All the recruited subjects were female and between the age range of 20-70 years. The patients were the diagnosed confirmed cases of breast cancer without any viral infections like HCV and HPV. Patients did not have adenocarcinoma or other forms of cancers.

### Data Collection

The data collection was based social information of the subjects which include marital status, parity and number of children. Family history with disease history was also collected from each patient which covers types of breast cancer, types of receptors, nature of breast cancer, menopausal status and staging of breast cancer.

### Statistical Analysis

Frequency distribution and mean ± SEM of age, family history, marital status, parity, age at first birth, number of children, height, weight and BMI was calculated. Patients were classified on the basis of marital status, family history and parity. Grouping of patients was done by their age groups, stage of cancer, groups of number of children and groups of age at first birth.

Furthermore, Chi-Square was used for association analyses between age groups with stages of breast cancer and groups of age at first birth with groups of number of children. All these statistical procedures were done by SPSS version 17.0.

## RESULTS

Both groups did not differ in terms of socio demographic and anthropometric features. Table-I. Most of the patients did not have family history of cancer in both groups. Distribution pattern of parity for married cases was calculated. It showed that parous cases (96.0% and 89.3%) predominate the nulliparous cases (4.0% and 10.7%). As described in Table-II most of the patients were below 45 years of age and had stage II breast cancer. Among the parous cases; age of patient at first birth and average number of children was calculate. It was found that maximum number of patients bear first child in the age range of 16 – 23 years with an average of 1 – 5 children.

**Table-I:** Sociodemographic and Anthropometric Features of Triple Negative and Triple Positive Breast Cancer.

Parameter	Triple Negative	Triple Positive
***Sociodemographic Features***		
***Age***		
Mean±SEM	46.517±1.195	45.694±0.809
***Family History***		
No family history	104 (77.6%)	70 (80.5%)
Family history of breast cancer	18 (13.4%)	11 (12.6%)
Family history of other cancers	12 (9.0%)	6 (6.9%)
***Marital Status***		
Married	125 (93.3%)	84 (96.6%)
Single	9 (6.7%)	3 (3.4%)
***Parity***		
Nulliparous	5 (4.0%)	9 (10.7%)
Parous	120 (96.0%)	75 (89.3 %)
***Age at first birth***		
	19.64±0.788	17.49±1.022
***Number of Children***		
	3.81±0.232	3.98±0.299
***Anthropometric Features***		
Height (meters)	1.58±0.0031	1.56±0.0184
Weight (kilograms)	70.52±1.098	70.74±1.445
BMI (kg/m^2^)	28.18±0.419	28.28±0.553

**Table-II:** Classification of Clinical Features:

Parameter	Triple Negative	Triple Positive
***Distribution of Patients among Different Age Groups***		
<45 (PreMenopausal)	76 (56.7%)	45 (51.7%)
45-50 (Menopausal)	39 (29.1%)	23 (26.4%)
50< (PostMenopausal)	19 (14.2%)	19 (21.8%)
***Distribution of Stages of Breast Cancer***		
Stage I	16 (11.9%)	10 (11.5%)
Stage II	67 (50.0%)	45 (51.7%)
Stage III	39 (29.1%)	28 (32.2%)
Stage IV	12 (9.0%)	4 (4.6%)
***Distribution of Cases into Different Groups of Age at First Birth***		
<15	11 (9.2%)	7 (9.3%)
16-23	70 (58.3%)	44 (58.7%)
24-31	31 (25.8%)	21 (28%)
32<	8 (6.7%)	3 (4.0%)
***Distribution of Number of Children into Different Groups***		
0 (No successful birth)	7 (5.8 %)	1 (1.3%)
1-5	81 (67.5%)	41 (54.7%)
6-10	30 (25.0%)	22 (29.3%)
>10	2 (1.7%)	11 (14.7%)

No significant association was found between menopausal status and stages of both triple negative and triple positive breast cancer groups. Table-III. Significant association (*χ*^2^ 79.796, *p* < 0.001) was found between the age groups of first birth and number of children in triple negative cases whereas, in triple positive cases no significant association was found between them. (Table-IV).

**Table-III:** Association Analyses: Age Groups (Menopausal Status) with Stages of Breast Cancer in Triple Negative and Triple Positive Breast Cancer.

Stages of Breast Cancer	Age Groups (Menopausal Status)							
	Triple Negative				Triple Positive			

	Pre-menopausal	Menopausal	Post-menopausal	Total	Pre-menopausal	Menopausal	Post-menopausal	Total
Stage I	12	3	1	16	4	5	1	10
Stage II	35	25	7	67	27	7	11	45
Stage III	23	7	9	39	14	9	5	28
Stage IV	6	4	2	12	0	2	2	4
	76	39	19	134	45	23	19	87
Pearson Chi Square	χ^2^12.371		p value 0.261		χ^2^13.937		p value 0.083	

**Table-IV:** Association Analyses: Groups of Age at First Birth with Groups of Number of Children in Triple Negative and Triple Positive Breast Cancer.

Groups of Number of Children	Groups of Age at First Birth									
	Triple Negative					Triple Positive				

	<15	16-23	24-31	32<	Total	<15	16-23	24-31	32<	Total
0 (No successful births)	7	0	0	0	7	0	0	0	1	1
1-5	2	46	27	6	81	3	21	16	1	41
6-10	2	22	4	2	30	2	18	1	1	22
>10	0	2	0	0	2	2	5	4	0	11
	11	70	31	8	120	7	44	21	3	75
Pearson Chi Square	χ^2^79.796		p value 0.000			χ^2^10.640		p value 0.100		

## DISCUSSION

Demographic, medical and anthropometric features of breast cancer were analyzed to find their association in the development and pathogenesis of triple negative breast cancer and triple positive breast cancer.

Frequency distribution and data classification reveal that the sociodemographic, anthropometric and medical features of triple negative breast cancer and triple positive breast cancer are not significantly different from each other (Table-I). This pattern of distribution between them is evident from the fact that both the study groups are stratified from a single breast cancer population and share most of the characteristics in a similar pattern and distribution.

The current study showed that being an overweight, having higher BMI, sedentary lifestyle are some of the factors leading to hormonal imbalance with irregular reproductive cycle (Table-I). It has also been shown from the average higher BMI and average weight of both triple negative and triple positive breast cancer cases. As mentioned in Table-I, the BMI is around 29 which is considered as overweight and one of potential factor of hormonal imbalance leading to carcinogenesis in both triple positive and triple negative breast cancer. 7,8,10,11

Further association analyses were done within the groups separately and then compared. Table-II represents the distribution if triple negative breast cancer and triple positive breast cancer cases into different age groups, stages of breast cancer, groups of age at first birth and groups for number of children. Age of patients were classified into different groups with respect to their menopausal status as premenopausal, menopause and post menopause. Premenopausal cases predominate the distribution. Premenopausal age less than 45 years is the active reproductive phase and susceptible to hormonal imbalance. 11 Moreover, the distribution of patients into different stages showed that the highest number of patients were at stage II followed by stage III breast carcinoma in both triple negative and triple positive age groups. Lack of awareness, cultural shyness are social factors which lead to the delay in diagnosis and treatment and cause advancement in the stages of cancer.^5^,^6^

In Table-III association analyses of age groups with stages of breast cancer within each strata were performed. The *p* value of triple positive breast cancer is closer to level of significance i.e. *p*<0.05 as compare to triple negative breast cancer. Pre-menopausal age with stage II and stage III breast carcinoma is predominant in both groups. Hormonal imbalance has been proved to be one of the prognostic factors of triple positive breast cancer treatment. Pre-menopausal age is vulnerable to hormonal imbalance because of inactive lifestyle, being overweight and disturbed reproductive life. Therefore, pre-menopausal age has impact on the development of triple positive breast cancer at stage II in the presented study population, which can be increased by increasing the number of cases.^12^-^14^

First child bearing age or age at first birth and number of children each patient has had been classified into different groups in Table-IV. Only those patients are under consideration who were married and nulliparous or parous. Frequency distribution analysis revealed that in both groups most of the patients have children in the range of one to five which makes the average of ~3 as mentioned in Table-I. Early first child bearing age reduces the risk of breast cancer development. As the number of children increases the risk decreases.^15^

By the association analysis of groups of age at first birth with the groups of number of children, it was found that late age at first birth has significant effect in the outcomes and advancement of triple negative breast cancer.^16^

Childbearing reduces the risk of breast cancer development and higher number of full-term pregnancies provides protection against it.^17^,^18^ Delayed parity increases the risk of breast cancer and it is shown in Table-IV that triple negative breast cancer group has more number of patients in delayed parity (i.e. >32 year) with less number of children / full term pregnancies than triple positive breast cancer which increase their chances of developing breast cancer (*p*<0.001).^16^,^19^,^20^

## CONCLUSIONS

Delayed parity with low number of children increase the risk of breast cancer development. Higher BMI, hormonal imbalance and disturbed reproductive life is also one of the most important risk factors of breast cancer. These sociodemographic and reproductive risk factors are not only the risk effectors but also proved to be one of the potential aspects which help in disease prognosis, treatment and affect the overall survival rate of breast cancer.

## References

[1] Jacques F, Isabelle S, Rajesh D, Sultan E, Colin M, Marise R (2015). Cancer incidence and mortality worldwide:Sources, methods and major patterns in GLOBOCAN 2012. Int J Cancer.

[2] Sarwar MR, Saqib A (2017). Cancer prevalence, incidence and mortality rates in Pakistan in 2012. Cogent Medicine.

[3] Sohail S, Alam SN (2007). Breast cancer in Pakistan - awareness and early detection. J Coll Physicians Surg Pak.

[4] Bener A, Catan F, El Ayoubi HR, Acar A, Ibrahim WH (2017). Assessing breast cancer risk estimates based on the gail model and its predictors in qatari women. J Primary Care Community Health.

[5] Salman K, Zoucha R, Nawafleh H (2018). Understanding Jordanian women's values and beliefs related to breast cancer:a focused ethnography. J Transcultural Nursing.

[6] Bedi M, Devins GM (2016). Cultural considerations for South Asian women with breast cancer. J Cancer Survivorship.

[7] Maas P, Barrdahl M, Joshi AD, Auer PL, Gaudet MM, Milne RL (2016). Breast cancer risk from modifiable and non-modifiable risk factors among white women in the united states. JAMA Oncol.

[8] Montaruli A, Patrini P, Roveda E, Carandente F (2012). Physical activity and breast cancer. Sport Sci Health.

[9] Oh H, Eliassen AH, Beck AH, Rosner B, Schnitt SJ, Collins LC (2017). Breast cancer risk factors in relation to estrogen receptor, progesterone receptor, insulin-like growth factor-1 receptor, and Ki67 expression in normal breast tissue. NPJ Breast Cancer.

[10] Nelson SH, Marinac CR, Patterson RE, Nechuta SJ, Flatt SW, Caan BJ (2016). Impact of very low physical activity, BMI, and comorbidities on mortality among breast cancer survivors. Breast Cancer Res Treat.

[11] Rae LK, Cheol HI, Do HK, Jinhyung J, Hae SM (2018). Waist circumference and risk of breast cancer in Korean women:A nationwide cohort study. International J Cancer.

[12] Hvidtfeldt UA, Tjonneland A, Keiding N, Lange T, Andersen I, Sorensen TIA (2015). Risk of breast cancer in relation to combined effects of hormone therapy, body mass index, and alcohol use, by hormone-receptor status. Epidemiology.

[13] Rosner B, Eliassen AH, Toriola AT, Hankinson SE, Willett WC, Natarajan L (2015). Short-term weight gain and breast cancer risk by hormone receptor classification among pre- and postmenopausal women. Breast Cancer Res Treat.

[14] Shin S, Saito E, Inoue M, Sawada N, Ishihara J, Takachi R (2016). Dietary pattern and breast cancer risk in Japanese women:the Japan Public Health Center-based prospective Study (JPHC Study). Brit J Nutr.

[15] Van Erkelens A, Derks L, Sie AS, Egbers L, Woldringh G, Prins JB (2017). Lifestyle risk factors for breast cancer in BRCA1/2-mutation carriers around childbearing age. J Genet Counsel.

[16] Newcomb PA, Trentham-Dietz A, Hampton JM, Egan KM, Titus-Ernstoff L, Andersen SW (2011). Late age at first full term birth is strongly associated with lobular breast cancer. Cancer.

[17] Lord SJ, Bernstein L, Johnson KA, Malone KE, McDonald JA, Marchbanks PA (2008). Breast cancer risk and hormone receptor status in older women by parity, age of first birth, and breastfeeding:a case-control study. Cancer Epidemiol Biomarkers Prev.

[18] Britt K, Ashworth A, Smalley M (2007). Pregnancy and the risk of breast cancer. Endocr Relat Cancer.

[19] Phipps AI, Chlebowski RT, Prentice R, McTiernan A, Wactawski-Wende J, Kuller LH (2011). Reproductive history and oral contraceptive use in relation to risk of triple-negative breast cancer. J Natl Cancer Inst.

[20] Kaminska M, Ciszewski T, Lopacka-Szatan K, Miotła P, Staroslawska E (2015). Breast cancer risk factors. Przegla-d Menopauzalny/Menopause Review.

